# Bacillus Calmette-Guerin (BCG) induces superior anti-tumour responses by Vδ2^+^ T cells compared with the aminobisphosphonate drug zoledronic acid

**DOI:** 10.1093/cei/uxac032

**Published:** 2022-04-11

**Authors:** J Fenn, L A Ridgley, A White, C Sarfas, M Dennis, A Dalgleish, R Reljic, S Sharpe, M Bodman-Smith

**Affiliations:** 1 Institute for Infection and Immunity, St. George's, University of London, London, UK; 2 NIHR Health Protection Research Unit in Respiratory Infections, National Heart and Lung Institute, Imperial College London, London, UK; 3 UK Health Security Agency, Porton Down, UK

**Keywords:** cytotoxic T cells, human, T cells, tumour immunology, bacterial

## Abstract

Vδ2^+^ T cells can recognize malignantly transformed cells as well as those infected with mycobacteria. This cross-reactivity supports the idea of using mycobacteria to manipulate Vδ2^+^ T cells in cancer immunotherapy. To date, therapeutic interventions using Vδ2^+^ T cells in cancer have involved expanding these cells *in* or *ex vivo* using zoledronic acid (ZA). Here, we show that the mycobacterium Bacillus Calmette–Guérin (BCG) also causes Vδ2^+^ T-cell expansion *in vitro* and that resulting Vδ2^+^ cell populations are cytotoxic toward tumour cell lines. We show that both ZA and BCG-expanded Vδ2+ cells effectively killed both Daudi and THP-1 cells. THP-1 cell killing by both ZA and BCG-expanded Vδ2+ cells was enhanced by treatment of targets cells with ZA. Although no difference in cytotoxic activity between ZA- and BCG-expanded Vδ2+ cells was observed, BCG-expanded cells degranulated more and produced a more diverse range of cytokines upon tumour cell recognition compared to ZA-expanded cells. ZA-expanded Vδ2+ cells were shown to upregulate exhaustion marker CD57 to a greater extent than BCG-expanded Vδ2+ cells. Furthermore, ZA expansion was associated with upregulation of inhibitory markers PD-1 and TIM3 in a dose-dependent manner whereas PD-1 expression was not increased following expansion using BCG. Intradermal BCG vaccination of rhesus macaques caused *in vivo* expansion of Vδ2^+^ cells. In combination with the aforementioned *in vitro* data, this finding suggests that BCG treatment could induce expansion of Vδ2^+^ T cells with enhanced anti-tumour potential compared to ZA treatment and that either ZA or BCG could be used intratumourally as a means to potentiate stronger anti-tumour Vδ2^+^ T-cell responses.

## Teaser

Vd2+ gamma-delta T cells have been shown *in vitro*to elicit potent cytolytic responses toward tumour cells, suggesting the potential for these responses to be manipulated in cancer immunotherapy. We show that heat-killed Bacillus Calmette-Guerin (HKBCG) promotes the expansion of Vd2+ T cells both *in vitro*and *in vivo*. These HKBCG-expanded cells exhibited enhanced degranulation and cytokine production in tumour cells compared to cells expanded using bisphosphonates.

## Introduction

Vδ2^+^ T cells are the predominant subset of γδ T cells in human peripheral blood. They express TCR δ chains that use the Vδ2^+^ arrangement and predominantly pair expression of these δ chains with the Vγ9 γ chain [1]. This Vδ2^+^ TCR arrangement enables these cells to recognize small phosphate antigens (pAgs) via the endogenous pseudo-presenting molecule butyrophilin 3A1 (BTN3A1) [2–4]. Endogenous pAgs occur under homeostatic conditions as intermediates of the mevalonate metabolic pathway which is involved in the production of cholesterol and in the prenylation of proteins. Dysregulation of this pathway occurs in malignantly transformed cells and can result in the accumulation of mevalonate pathway intermediates such as isopentenyl pyrophosphate (IPP) [5]. This accumulation can lead to the recognition of tumour cells by Vδ2^+^ cells, supporting a role for Vδ2^+^ cells in immunosurveillance against tumours [6, 7].

Exogenous pAgs are produced by a range of pathogens, including plasmodia and bacteria, and can also be detected by Vδ2^+^ cells via their Vδ2^+^ TCR. Mycobacteria, for example, have been shown to use the MEP/DOXP pathway (a prokaryotic analog of the mevalonate pathway) in which the pAg (E)-4-hydroxy-3-methyl-but-2-enyl pyrophosphate (HMBPP) is produced as an intermediate. HMBPP displays a 30 000-fold higher potency than IPP, inducing proliferation and cytokine production by Vδ2^+^ cells even at the picomolar range [8]. Bacterially derived HMBPP has been shown to render human cells susceptible to recognition and destruction by Vδ2^+^ cells. As such, Vδ2+ cells have been implicated in immune responses to HMBPP-producing pathogens including *Mycobacterium tuberculosis*, *Listeria monocytogenes*, and *Salmonella enterica* [9–11]. The ability of Vδ2^+^ cells to recognize both bacterially infected and malignantly transformed cells raises the question of whether pAg-producing bacteria could be used to manipulate Vδ2^+^ cell responses in a manner that could facilitate enhanced anti-tumour Vδ2^+^ cell activity.

The aminobisphosphonate drug Zoledronic acid (ZA) is used routinely in the treatment of osteoporosis and bone degeneration associated with the development of bone metastases [11, 12]. It may also have the potential as an immunotherapeutic agent. In the context of γδ T cells, ZA has been shown to inhibit the mevalonate pathway at the level of farnesyl pyrophosphate synthase (FPPS), leading to an accumulation of IPP which results in normally Vδ2^+^ cell-resistant tumour cells becoming susceptible to recognition and killing by Vδ2^+^ cells [7]. ZA administration both *in vitro* and *in vivo* has been shown to lead to the activation and proliferation of potentially tumour-reactive Vδ2^+^ cells. Both methods of Vδ2^+^ cell expansion have been evaluated in clinical trials [13–20].

Bacillus Calmette-Guérin (BCG) is routinely used in the treatment of non-invasive bladder cancer and has also been used intralesionally in the treatment of melanoma [21–23]. In both cases, treatment has been associated with favourable clinical responses in cancer patients that appear to be associated with the infiltration of various types of immune cells into the site where BCG was administered [24–26]. Vδ2^+^ cells have been shown to be capable of lysis of mycobacterial infected cells, making them candidate mediators of anti-tumour activity induced by BCG treatment [27, 28]. Supporting this speculation is evidence of infiltration of activated, IFNγ^+^ Vδ2^+^ cells into BCG-treated melanoma lesions where their presence was associated with lesion regression [29].

In this study, we investigate potential mechanisms by which BCG could be used to potentiate anti-tumour Vδ2^+^ cell responses. We show that BCG can be used to induce the proliferation of Vδ2^+^ cells in PBMCs and that the resulting Vδ2^+^ cell populations produced a more diverse and higher magnitude cytokine response to tumour cells than ZA-expanded Vδ2^+^ cells. The failure of ZA-expanded cells to respond optimally to tumour target cells was associated with upregulation of exhaustion and inhibitory markers CD57 and PD-1 respectively. *In vivo* studies using the rhesus macaque model of BCG vaccination show that intradermal BCG vaccination is sufficient to induce expansion of Vδ2^+^ cells in the peripheral blood. Together, these results suggest that BCG vaccination could be a viable way of promoting the expansion of Vδ2^+^ cells with enhanced anti-tumour potential and furthermore that BCG or ZA could be used to target Vδ2^+^ cell responses toward tumour cells.

## Materials and methods

### PBMC isolation

PBMCs were isolated from anonymized leukocyte cones which were acquired from NHS Blood and Transplant, St. George’s, University of London (NHSBT) under ethical approval SGREC16.0009. Contents of leukocyte cones were diluted at approximately 1:10 in RPMI 1640 media (Sigma) supplemented with 10% foetal bovine serum (FBS) (Sigma) and PBMCs were isolated by density gradient centrifugation using Histopaque-1077 (Sigma). Residual erythrocytes were lysed using RBC lysis buffer (BioLegend) and platelets were removed by repeated centrifugation at 200g. PBMCs were re-suspended in a freezing medium (45% RPMI-1640, 45% FBS, 10% DMSO) and stored for 2 days at −80°C before being transferred for storage in liquid nitrogen.

### Cell culture

#### Tumour cell culture

Daudi and THP-1 cells were acquired from the European Collection of Authenticated Cell Cultures (ECACC) (Public Health England, Porton Down). Cell lines were maintained by culturing in RPMI 1640 medium containing 10% FBS at 37°C with 5% CO_2_ and were passaged every 2–4 days to maintain recommended cell densities. Passage numbers were universally kept below 20.

#### BCG culture

BCG Pasteur strain and GFP-expressing BCG Pasteur strain were kind gifts of Dr Rajko Reljic (St. George’s, University of London). Bacterial cells were cultured in 7H9 media (Becton Dickinson) supplemented with 10% Middlebrook ADC enrichment (Sigma) and 0.05% Tween 80 (Sigma) and grown in a shaking incubator at 37 °C for 4 weeks after which cells were washed and re-suspended in 15% glycerol and 85% 7H9 Liquid medium and frozen at −80 °C in cryogenic vials (Nalgene). Prior to use in assays, bacteria were enumerated using a colony-forming unit counting method in which bacteria were inoculated onto agar plates containing 7H11 media (Becton Dickinson) supplemented with 10% Middlebrook OADC enrichment (Sigma). The number of visible colonies was determined after 3 weeks of culture at 37 °C. In some instances, where indicated, BCG was heat-killed at 80 °C for 30 min prior to culture with PBMCs.

#### BCG and ZA expansion of Vδ2^+^ cells

5 × 10^6^ thawed PBMCs/mL were stimulated in round-bottomed 96 well plates with 15 ng/mL IL-2 (R&D systems) in combination with either 0.5, 1, 5, or 10 μM ZA (Sigma) or 2 × 10^4^ CFU BCG/well. Every 2–3 days, 100 μL of culture media was replaced with 100 μL fresh media containing 30 ng/mL IL-2. Cells were cultured for 12 days before use in downstream applications. In experiments where isolated Vδ2^+^ cells were used. Expanded cells were re-suspended in MACS buffer (PBS, 0.5% BSA, 2mM EDTA) and isolated using the γδ T-cell negative selection kit—130-092-892 (Miltenyi Biotec) according to the manufacturer’s instructions.

### Flow cytometry

#### Surface and intracellular staining

Cells were collected, washed using FACS buffer (PBS (Sigma) containing 1% BSA (Sigma), 0.1% sodium azide (NaN_3_) (Sigma), 2 mM ethylenediaminetetraacetic acid (EDTA) (Sigma)) and stained for 15 min at room temperature with fluorescently conjugated antibodies at manufacturer recommended concentrations. Alternatively, for intracellular staining experiments, cells were re-suspended in 500 μL fixation buffer (BioLegend) and incubated for 20 min before the addition of 500 μL 1× permeabilization wash buffer (BioLegend) and 2 further wash steps with permeabilization wash buffer. Permeabilized cells were stained for 20 min at room temperature with fluorescently conjugated antibodies at manufacturer recommended concentrations and washed in permeabilization wash buffer. All stained cells were washed with FACS buffer before being re-suspended in 300 μL Cellfix (BD) and acquired using a BD LSRII flow cytometer or LSR Fortessa X20 (both BD Biosciences). The following antibodies were used: anti-CD3-Alexafluor 700 (OKT3), anti-CD3-PerCP (OKT3), antiCD69-FITC (FN50), anti-CD107b-PE (H4B4), anti-CD16-PE (3G8), anti-CD57-PE-Cy7 (HNK-1), anti-NKG2D-APC (1D11), anti-granulysin-PE (DH2) all from BioLegend; anti-Vδ2-PerCP-Vio700 (REA771), anti-CD56-VioBright FITC (AF12-7H3), anti-Vδ2-PE (123R3), anti-IFNγ-APC-Vio770 (45-15), anti-perforin-APC (δG9)—all from Miltenyi Biotec and anti-Vγ9-FITC (7A5) from Thermo-Fisher Scientific. Data were analysed using FlowJo v10 (BD).

#### CD107b mobilization assay

A solution containing 5 μL FITC-conjugated CD107b antibody, 5 μg/mL Brefeldin A, 2 μM Monensin (all BioLegend), and 5 μL media was added directly to 200 μL effector: target co-cultures at 10 μL per well 15 min after initiation of co-culture. Cells were incubated for 4 h before being harvested and stained using the protocol described in the ‘Flow cytometry’ section. Phorbol 12-myristate 13-acetate (PMA) (25 ng/mL) and ionomycin (I) (1 μg/mL) were added to positive control wells prior to the addition of monensin and brefeldin.

### Cytotoxicity assay

Tumour target cells were prepared by the overnight culture of 6 × 10^5^ cells/mL in the presence of 50 μM ZA or BCG at a multiplicity of infection (MOI) of 1. These cells were then labelled for 15 min with Cell Trace Far Red (Life technologies) in PBS at manufacturer recommended concentrations, washed twice with RPMI 1640 media supplemented with 10% FBS, and added to round bottom 96 well plates at 5 × 10^4^ cells per well. Expanded, isolated effector cells were added to target cells at a 1:1 effector to target ratio. Cells were co-cultured for 18 h before being labelled with live/dead red stain (Life technologies) in PBS for 20 min in the dark at room temperature and acquired using BD LSRII flow cytometer. The specific killing was calculated by subtracting the frequency of dead cells in ‘target alone’ conditions from the frequency of dead cells in co-culture conditions.

### Confocal microscopy

Growing THP-1 cells were washed in culture media and re-suspended at 1 × 10^6^ cells/mL. The cell suspension was transferred to black flat-bottomed 96 well plates (Corning) at 100 μL per well to give 1 × 10^5^ cells per well. GFP-expressing BCG was thawed, re-suspended to give 1 × 10^6^ CFU/mL, or 5 × 10^6^ CFU/mL as indicated and added to plates at 100 μL per well to give THP-1: BCG bacilli ratios of 1:1 and 1:5, respectively. After 24 h, cells were washed twice with PBS before being fixed by re-suspension in 4% paraformaldehyde (PFA; Sigma) in the dark at room temperature for 10 min. Cells were washed twice with PBS and resuspended in PBS containing 1 μg/mL 4ʹ,6-diamidino-2-phenylindole (DAPI). Labelled cells were imaged using a 40× CFI S Plan Fluor ELWD objective lens of a Nikon A1R confocal microscope. A 405 nm laser was used to detect DAPI fluorescence (displayed in blue) and 488 nm laser was used to detect GFP fluorescence (displayed in green). NIS Elements version 4.2 software was used to acquire and analyse data.

### Legendplex assay

A human CD8/NK Panel (BioLegend Cat. No. 740267) multi-analyte assay was used to detect proteins secreted during Vδ2^+^ cell-tumour cell co-culture. The kit was used according to the manufacturer’s instructions. Specifically, 100 μL supernatants were collected from γδ T-cell—tumour cell co-cultures after 24 h and frozen. Prior to use, supernatants were thawed and centrifuged at 1400g for 5 min to remove cell debris. Supernatant (25 μL) plus assay buffer (25 μL) were added to each well of a 96-well ‘V’ bottomed plate. A set of eight protein standards were prepared by serial dilution of a cocktail consisting of known concentrations of the recombinant forms of the 13 proteins detected by this kit in assay buffer. Aliquots of these standards (25 μL) plus culture medium (25 μL) were added to ‘standard’ wells of the 96 well plate in duplicate. To both sample wells and standard wells, 25 μL each of pre-mixed beads and detection antibodies were added before incubating in the dark on a plate shaker at 600rpm at room temperature for 2 h. PE-streptavidin (25 μL) was added before incubating on a plate shaker at 600 rpm for 30 min. Plates were washed twice by centrifugation at 1000g for 5 min. Samples were transferred to FACS tubes and immediately acquired using a BD LSRII flow cytometer using recommended data acquisition templates provided by BioLegend. FCS files were exported and analysed using LEGENDplex data analysis software.

### Macaque vaccination and sampling

All macaque studies were conducted in animals housed in compatible social groups and in accordance with the Home Office (UK) Code of Practice for the Housing and Care of Animals Used in Scientific Procedures (1989) and the Guidelines on Primate Accommodation, Care and Use of the National Committee for Refinement, Reduction and Replacement (NC3Rs), issued in August 2006. All protocols involving animals were approved by the Ethical Review Body of the Public health England, Porton, UK and were authorized under an appropriate UK Home Office project license. Animals were sourced from an established, closed UK breeding colony, none of the animals had been used previously for experimental procedures [30]. The absence of previous exposure to mycobacterial antigens was confirmed by tuberculin skin test as part of colony management procedures and by screening for IFN-γ ELISpot (MabTech, Nacka. Sweden) responses to tuberculin-PPD (purified protein derivative) (SSI, Copenhagen, Denmark), and pooled 15-mer peptides of ESAT6 and CFP10 (Peptide Protein Research LTD, Fareham, UK).

Housing pens were designed to allow access to indoor and outdoor environments and measured approximately 20 m^2^ by 2.5 m high for indoor, and 37.8 m^2^ by 3.7 m high for outdoor-enclosures. Pens were constructed with a range of high-level observation balconies and a floor of deep litter was provided in internal enclosures to allow foraging. Additional environmental enrichment was afforded by the provision of toys, swings, climbing apparatus, and feeding puzzles for stimulation. In addition to standard old-world primate pellets, the diet was further supplemented with a selection of fresh vegetables and fruit. For each procedure, sedation was applied by intramuscular injection with ketamine hydrochloride (10 mg/kg) (Ketaset, Fort Dodge Animal Health Ltd, Southampton, UK).

Six of a cohort of 12 (one male, 11 female) rhesus macaques (*Macaca mulatta*) of Indian origin aged between 14 and 16 years were immunized intradermally in the upper left arm with 100 μL BCG, Danish strain 1331 (SSI, Copenhagen, Denmark). The BCG vaccine was prepared and administered according to the manufacturer’s instructions for the preparation of a vaccine for administration to human adults; this was done by adding 1 mL Saunton’s medium as the diluent to a vial of vaccine to give a suspension of BCG at an estimated concentration of 2 × 10^6^ to 8 × 10^6^ CFU/mL [31]. Ten millilitre blood samples were collected 4 and 6-weeks post-vaccination. Peripheral blood mononuclear cells (PBMCs) were isolated from heparin-anticoagulated blood by Ficoll–Hypaque Plus (GE Healthcare, Buckinghamshire, UK) density-gradient separation using standard procedures as previously described [32].

### ELIspot

An IFN-γ enzyme-linked immunosorbent spot (ELISpot) assay was used to determine the numbers of IFN-γ producing mycobacterium-specific T cells in PBMCs using a human/monkey IFN-γ kit (Mabtech) as described previously [32]. In brief, PBMCs were cultured with 10 μg/mL purified protein derivative (PPD) (SSI, Copenhagen), or without antigen, in triplicate, and incubated for 18 h. Phorbol 12-myristate (100 ng/mL) (Sigma) and ionomycin (1 μg/mL) (CN Biosciences) were used as a positive control. After culture, spots were developed according to the manufacturer’s instructions. Determinations from triplicate tests were averaged. Data were analysed by subtracting the mean number of spots in medium-only control wells from the mean counts in antigen-stimulated wells [31].

## Results

### BCG causes the activation and proliferation of Vδ2^+^ cells

**Figure 1:**
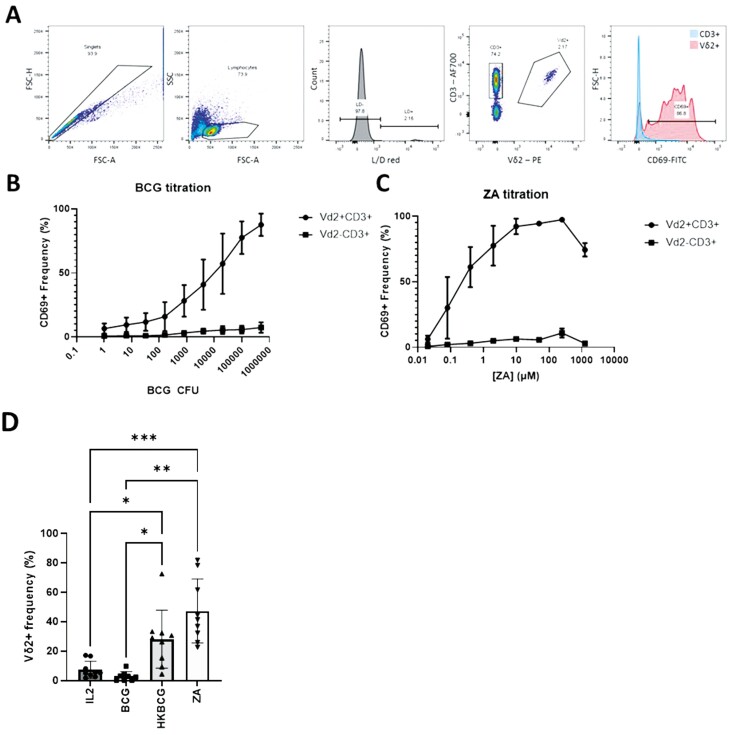
PBMCs were thawed and plated at 5 × 10^5^ cells per well of round-bottomed 96 well plates and stimulated with ZA or BCG at the indicated concentrations for 12 days. Vδ2+ cells responses were assessed using flow cytometry with CD69 staining as a marker of activation and expansion determined by frequencies of Vδ2+ cells as a percentage of live cells. **A**. Gating strategy used to identify CD69 positive CD3+Vδ2− and CD3+Vδ2+ cells. Example cells from one donor stimulated with 2 × 10^4^ CFU BCG. **B**. Stimulation assay conducted for a range of BCG CFU numbers (*N* = 5. Bars represent means ± SD). **C**. Similar assays were conducted using a range of ZA concentrations (*N* = 3. Bars represent means ± SD). **D**. Vδ2+ cell frequencies were assessed in PBMCs following 12 days under a number of stimulation conditions including 15 ng/mL IL-2 alone, 15 ng/mL IL-2 with 2 × 10^4^ CFU BCG or heat-killed BCG or 1 µM ZA based on results shown in 2.1 B and C. (*N* = 9. Bars represent means ± SD. Repeated-measures ANOVA with Geisser–Greenhouse correction and Tukey’s multiple comparison test *P < .05, **P < .01, ***P < .001.)

To address the hypothesis that BCG-primed Vδ2^+^ cells have anti-tumour activity, initial experiments sought to determine the effect of BCG on Vδ2^+^ cells in terms of activation state and induction of proliferation. Isolated PBMCs were treated with BCG at a range of CFU values or ZA at a range of concentrations as a control (based on extensive evidence that ZA induced Vδ2^+^ cell activation and proliferation). Activation of Vδ2^+^ cells and CD3^+^Vδ2^-^ cells was assessed using CD69 expression (Fig. 1A). Results show that both BCG and ZA cause activation of Vδ2^+^ cells in a dose-dependent manner (Fig. 1B and C). It was observed that at the highest tested CFU values, BCG appeared to induce cell death as assessed by light microscopy and shift in the FSC SSC characteristics of cells as determined by flow cytometry. Similarly, whilst ~100% of Vδ2^+^ cells were consistently activated by stimulation at 50 and 250 μM ZA, fewer Vδ2^+^ cells were activated at 1000 μM—likely due to the toxicity of the drug at this high concentration (Fig. 1C).

Following 12 days of stimulation with BCG and ZA, PBMCs were stained for expression of Vδ2^+^ TCR and CD3 enabling Vδ2^+^ and T-cell populations to be gated and Vδ2^+^ cell expansion to be assessed. Vδ2^+^ cell frequencies from 9 donors stimulated with IL-2 alone or IL-2 in combination with 2 × 10^4^ CFU BCG, heat-killed BCG, or 1 µM ZA. The concentration of ZA chosen induced similar levels of CD69 expression compared to BCG and in addition to induction of CD69 stimulation, 1 µM ZA induced high frequencies of Vδ2^+^ cells compared to IL-2 alone (Fig. 1D). A high level of batch variability was seen with 2 × 10^4^ CFU BCG with Fig. 1D showing data obtained using a BCG batch that caused high amounts of cell death and lower frequencies of Vδ2^+^ cells. In comparison, heat-killed BCG was subject to lower levels of batch variation and consistently induced robust Vδ2^+^ cell expansions. HKBCG was therefore used preferentially for subsequent Vδ2^+^ cell expansions. HKBCG stimulation resulted in higher levels of Vδ2^+^ cell expansion compared to IL-2 alone, as did stimulation with 1 µM ZA (*P = .*025 and 0.001, respectively) (Fig. 1D).

### HKBCG-expanded Vδ2^+^ cells degranulate in response to tumour cells

**Figure 2:**
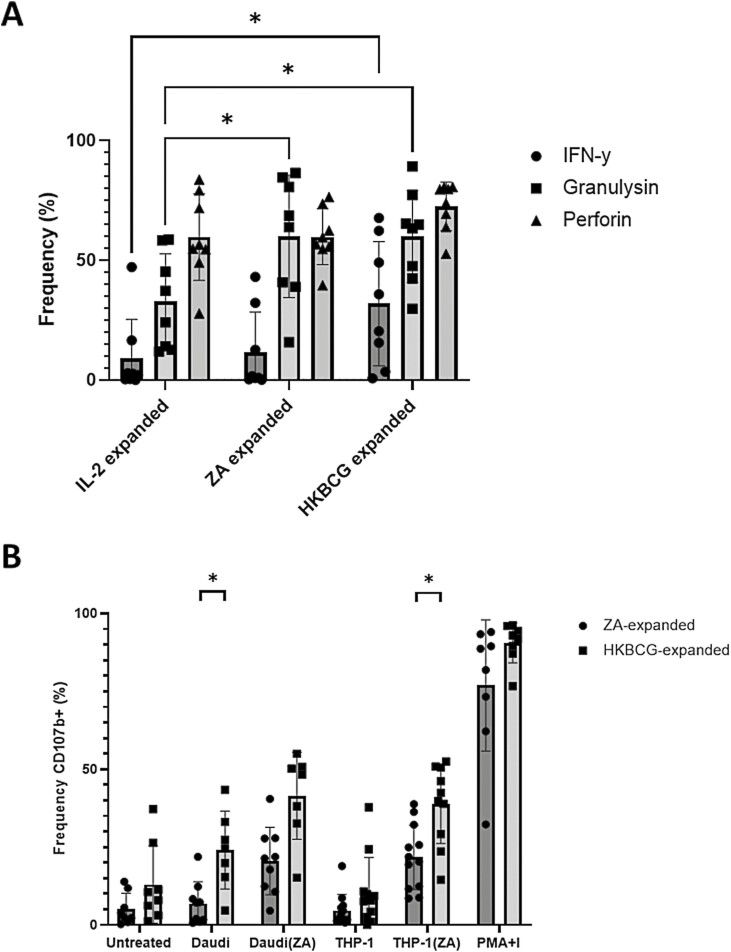
PBMCs from 8 donors were expanded using either 15 ng/mL IL-2 with 1 µM ZA or 2 × 10^4^ CFU heat-killed BCG. After 12 days cells were stained and analysed using flow cytometry. Vδ2+ cells were gated based on FSC SSC characteristics and double positivity for Vδ2-PerCPVio770 and CD3-AF700. **A**. ICS was used to determine post-expansion frequency of Vδ2+ cells expressing IFN-γ and cytolytic mediators granulysin and perforin. (*N* = 8. Bars represent means ± SD. Two-way ANOVA with full fit model and Tukey’s multiple comparison *P < .05). **B**. Daudi and THP-1 tumour cells were cultured overnight with or without 50 µM ZA. 5 × 10^5^ expanded PBMCs were co-cultured with tumour target cells at a 10:1 PBMC: tumour cell ratio for 4.5 h in the presence of anti-CD107b-FITC antibody, monensin, and brefeldin A. (*N* = 12. Bars represent means ± SD. multiple paired *T*-tests with Sidak–Bonferroni correction for multiple comparisons *P < .05.)

Vδ2+ cells are known to exert effector functions via the production of IFN-y, perforin, and granulysin [9, 33, 34]. Therefore, we measured the effect of HKBCG and ZA expansion on intracellular expression levels of these molecules. Both ZA and HKBCG expansion caused increased levels of intracellular granulysin expression compared to cells treated with IL-2 alone. In HKBCG-expanded, but not ZA-expanded Vδ2+ cells, intracellular IFN-y was also upregulated relative to IL-2 only control (Fig. 2A).

Having shown accumulation of effector molecules during expansion, we sought to compare the ability of HKBCG-expanded Vδ2+ cells to degranulate in response to tumour target cells to ZA-expanded Vδ2+ cells. HKBCG- or ZA-expanded PBMCs were cultured with tumour cells in the presence of BFA, monensin, and anti-CD107b-FITC. The results of these assays show that Vδ2^+^ cells from HKBCG-expanded cells degranulated more toward Daudi cells and ZA-treated THP-1 cells compared to their ZA-expanded counterparts (*P = .*020 and 0.022, respectively) (Fig. 2B). Additionally, the results show that ZA treatment of THP-1 cells renders them capable of inducing more degranulation in Vδ2^+^ cells.

### BCG infection of THP-1 cells does not significantly increase their susceptibility to Vδ2^+^ cell-mediated killing

**Figure 3:**
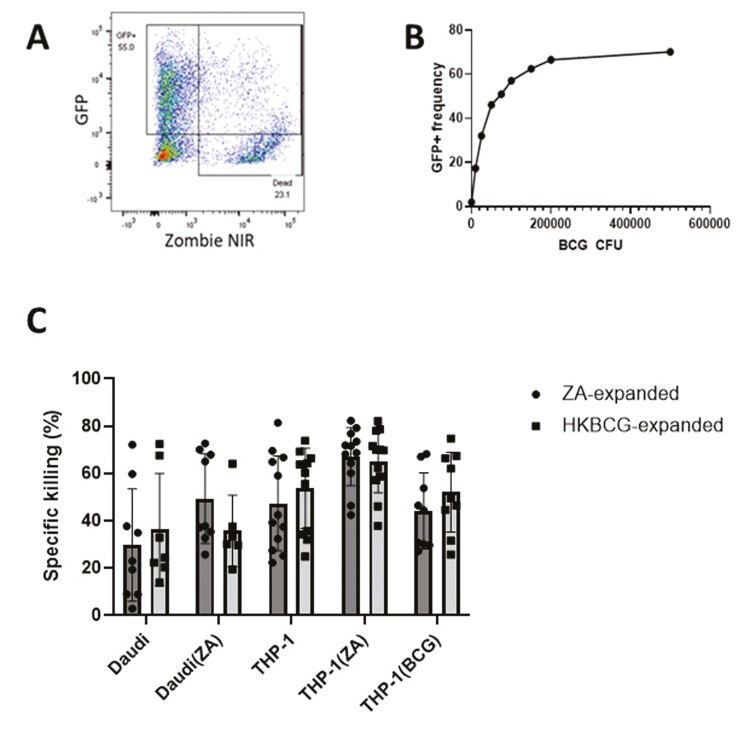
GFP transfected BCG was cultured overnight with 1 × 10^5^ THP-1 cells at a range of CFU values. **A**. A representative plot displaying the gating strategy used to identify live ‘infected’ THP-1 cells based on GFP and Zombie NIR fluorescence. **B**. The gating strategy displayed in A was applied to determine the percentage of successfully infected THP-1 cells after overnight culture with BCG at a range of CFU values. **C**. ZA-expanded Vδ2+ cells were isolated using MACS negative isolation and cultured overnight with 5 × 10^4^ CTFR-labelled target cells at a 3:1 effector: target cell ratio. Daudi and THP-1 target cells were cultured overnight either with or without 50 µM ZA or 1 × 10^5^ CFU BCG prior to initiation of co-culture. >5000 CTFR+ cells were acquired using a BD LSR Fortessa X20 flow cytometer. Target cells were gated based on CTFR positivity. The specific killing was calculated by subtracting the frequency of dead target cells (as determined by live/dead red positive staining) in target alone conditions from the frequency of dead cells in co-culture conditions. (*N* = 12. Bars represent means ± SD multiple paired *T*-tests with Sidak–Bonferroni correction for multiple comparisons.)

To determine whether BCG-infection could increase the susceptibility of tumour cells to lysis by Vδ2^+^ cells, THP-1 cells were infected with BCG and cultured in the presence of Vδ2^+^ cells. The BCG infection model used in this series of experiments was characterized using a GFP transformed strain of BCG. Flow cytometry assays showed that after culture in the presence of GFP^+^BCG, THP-1 cells increased in GFP fluorescence in a BCG dose-dependent manner (Fig. 3A and B). To confirm that this increase in fluorescence was associated with true BCG internalization, confocal microscopy was used to visualize the spatial distribution of bacilli within THP-1 cells (Supplementary Fig. S1). Rod-shaped GPF^+^ bacilli were detected inside THP-1 cells proximal to the nucleus. Infected cells commonly contained multiple bacilli, yet a proportion of cells remained uninfected even after 24 h, in agreement with the observation using flow cytometry that an upper limit existed in terms of the frequency of infected cells despite increasing BCG CFU values. Infection caused increasing THP-1 cell death with increasing BCG CFU (Supplementary Fig. S1). To limit the impact of this effect on future assays a compromise was struck between maximal infection efficacy and minimal cell death induction and an MOI of 1 was selected for assays involving infection of THP-1 cells with BCG.

It was hypothesized that BCG infection of THP-1 cells would be associated with increases in intracellular levels of bacterially derived pAg, HMBPP, and that this would cause Vδ2^+^ cells to elicit Vδ2 TCR-dependent killing of BCG-infected THP-1 cells (THP-1(BCG)). To test this hypothesis, ZA- and HKBCG-expanded Vδ2+ cells were enriched using MACS negative selection columns and co-cultured with tumour target cells, including BCG-infected THP-1 cells at a 3:1 ratio. Neither ZA- nor HKBCG-expanded Vδ2^+^ cells killed THP-1(BCG) cells at significantly higher levels than untreated cells (Fig. 3C). In addition to THP-1(BCG) target cells, expanded Vδ2+ cells were cultured with Daudi and THP-1 cells with and without ZA pre-treatment. In multiple pairwise comparisons, no significant differences between ZA- and HKBCG-expanded Vδ2+ cell killing of tumour cell targets were observed despite the prior observation of enhanced degranulation toward THP-1 cells by HKBCG-expanded Vδ2+ cells.

### ZA- and HKBCG-expanded Vδ2^+^ produce qualitatively and quantitatively different cytokine responses to tumour cells

**Figure 4:**
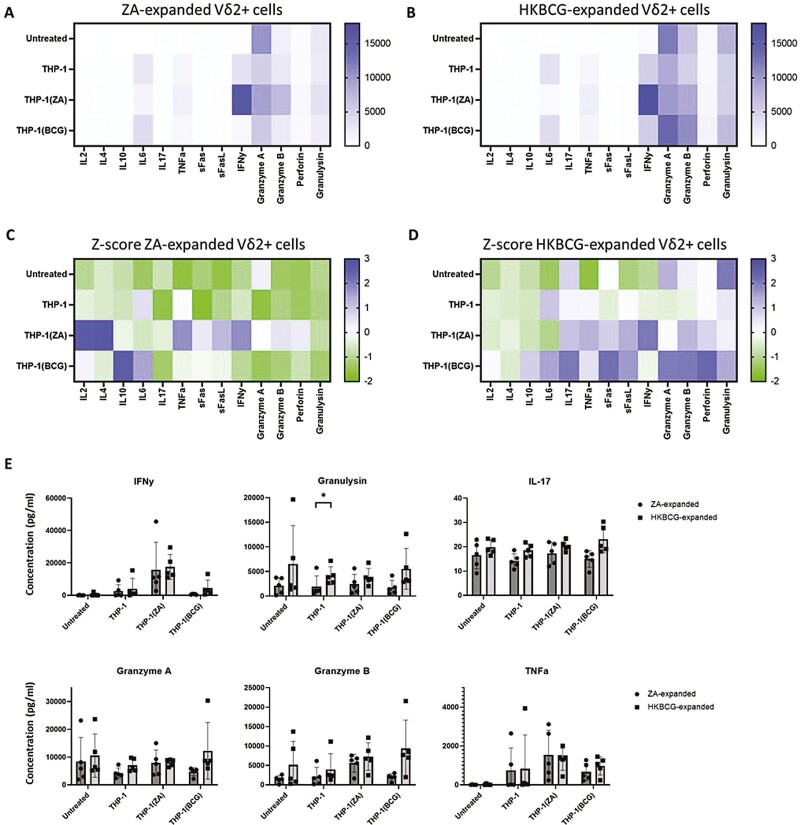
PBMCs from 5 donors were expanded using 15 ng/mL IL-2 with either 1 μM ZA or 2 × 10^4^ CFU heat-killed BCG/well for 12 days. 5 × 10^4^ ZA- or heat-killed BCG-expanded MACS-isolated Vδ2+ cells were co-cultured at a 1:1 effector:target ratio for 18 h with THP-1, THP-1(ZA), or THP-1(BCG) cells. Co-culture supernatants were used in Legendplex assays, with analysis performed using Biolegend LEGENDplex software. **A**–**B**. Heat maps displaying mean ng/mL analyte concentrations. **C**–**D**. Heat maps displaying *z*-scores for each analyte. **E**. Concentrations of IFN-γ, granulysin, IL-17, granzyme A, granzyme B, and TNF-α are shown for assays conducted using expanded and isolated Vδ2+ cells (*N*=5. Bars represent means ± SD multiple paired *T*-tests with Sidak–Bonferroni correction for multiple comparisons *P < .05.)

Supernatants from Vδ2+ cell cytotoxicity assays were collected and Legendplex assays were performed to compare the profiles of effector molecules released by ZA- and HKBCG-expanded Vδ2+ cells upon tumour cell recognition (Fig. 4A and B). To visualize differences in cytokine profiles between ZA- and HKBCG-expanded cells Z-scoring within individual analytes was performed and scored data was plotted as a heatmap (Fig. 4C and D). Compared to ZA-expanded Vδ2+ cells, HKBCG-expanded cells tended to release more cytolytic molecules including granzymes and granulysin in response to tumour cell co-culture, whereas ZA-expanded Vδ2+ cells tended toward relatively higher levels of Th2-associated cytokines. In comparisons of individual analytes, only production of granulysin in response to THP-1 cells was significantly higher in HKBCG-expanded cells as determined by multiple pairwise *T*-tests after correcting for multiple comparisons (*P = .*03) (Fig. 4E). Together this data characterizes HK-BCG-expanded cells as superior cytokine and cytolytic mediator producers compared to ZA-expanded cells.

### ZA expansion drives Vδ2+ cells toward an exhausted phenotype that is not observed following HKBCG expansion

**Figure 5:**
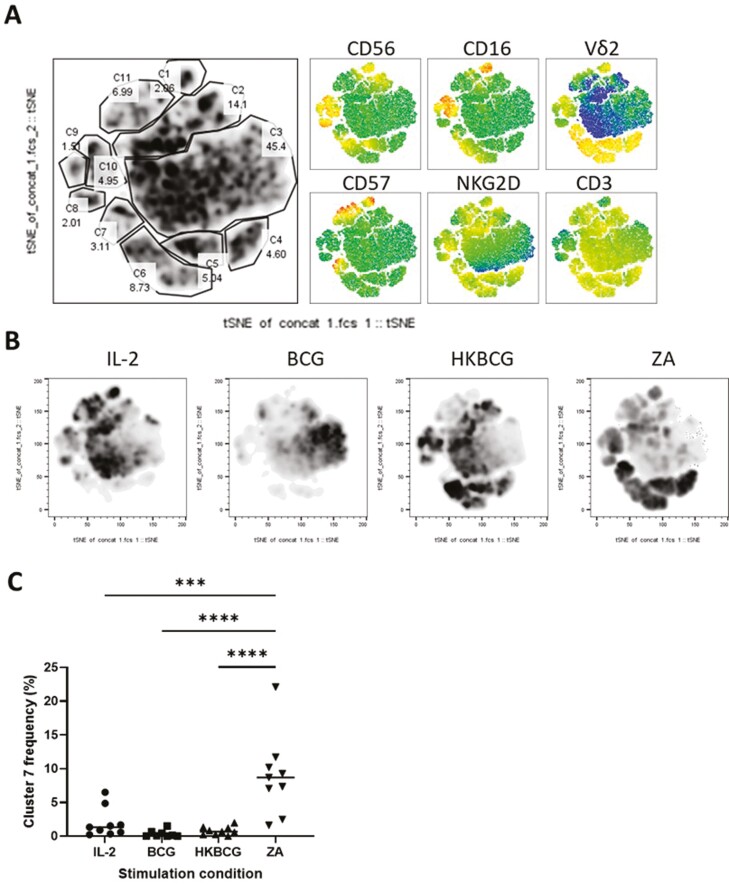
PBMCs from 9 donors were expanded using 15 ng/mL IL-2 with either 1 μM ZA or 2 × 10^4^ CFU heat-killed BCG/well. After 12 days cells were stained and analysed using flow cytometry. Vδ2+ cells were gated based on FSC SSC characteristics and double positivity for Vδ2 PerCPVio770 and CD3 AF700. Cells were assessed in terms of expression of CD16, CD56, CD57, and NKG2D. **A**. Equal numbers of events from each donor and each stimulation condition were concatenated and tSNE dimensionality reduction was performed. Cell clusters were manually identified and gated. Heat map overlays represent the level of expression of each marker independently. **B**. Density plots showing cell distribution by stimulation condition. Each plot represents concatenated data from 9 donors. **C**. Graph showing the frequency of cluster 7 (C7 in A) cells according to stimulation condition. (*N* = 9. Bars represent means ± SD. Repeated-measures ANOVA with Geisser–Greenhouse correction and Tukey’s multiple comparison ***P < .001, ****P < .0001.)

We next sought to investigate whether phenotypic differences could account for the increased cytokine-producing potential of HKBCG-expanded Vδ2^+^ cells. PBMCs from 9 donors were expanded using IL-2, BCG, HKBCG, and ZA for 12 days and the expression of CD3, Vδ2 TCR, CD16, CD56, NKG2D, and CD57 was determined using flow cytometry. Equal numbers of events from each donor and stimulation condition were concatenated and tSNE clustering was employed to agnostically identify clusters of cells that may be differentially associated with different expansion protocols (Fig. 5A). Striking differences in the distribution of tSNE clusters were observed between ZA- and HKBCG-expanded cells (Fig. 5B). Specifically, different frequencies of ‘cluster 7’ events were observed in ZA-expanded Vδ2+ cells compared to HKBGC-expanded cells (Fig. 5C). An ANOVA confirmed statistically significant differences in ‘cluster 7’ frequencies between ZA- and HKBCG-expanded cells (*P < .*0001). Using individual marker heatmap overlays, ‘cluster 7’ was identified as Vδ2+ cells expressing high levels of CD57. CD57 has been used as a marker of replicative senescence in Vδ2^+^ cells [35], suggesting that the differences in cytokine-producing potential between ZA- and HKBCG-expanded Vδ2+ cells could be associated with a highly differentiated state or functional exhaustion.

### ZA-expanded Vδ2+ cells express high levels of inhibitory checkpoint molecules

**Figure 6:**
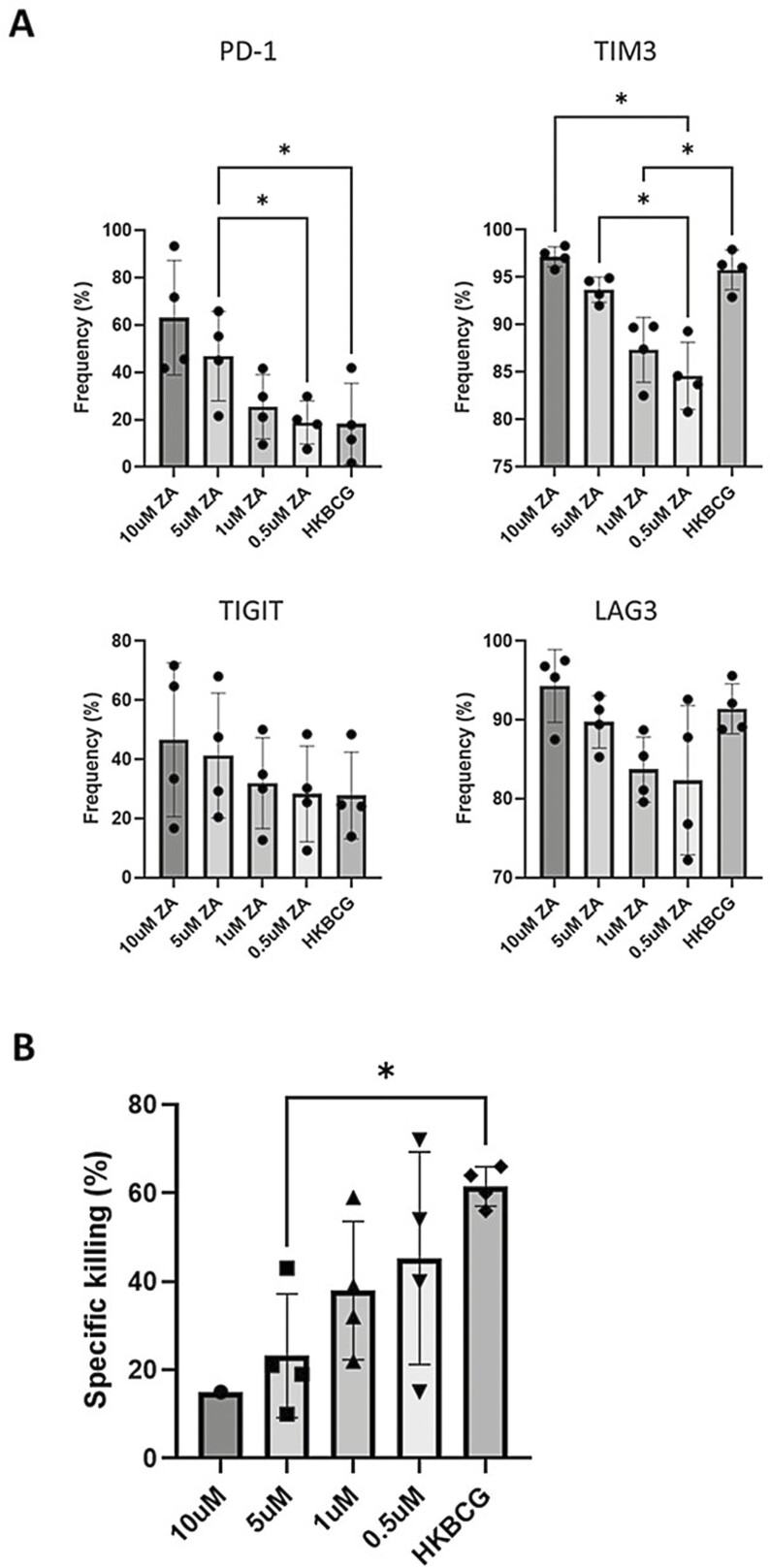
PBMCs from 4 donors were expanded using 15 ng/mL IL-2 with either 10, 5, 1, or 0.5 μM ZA or 2 × 10^4^ CFU heat-killed BCG/well. After 12 days cells were stained and analysed using flow cytometry. Vδ2+ cells were gated based on FSC SSC characteristics and double positivity for Vδ2 PerCPVio770 and CD3 AF700. **A**. Frequency of PD1, TIGIT, TIM3, and LAG3 positive Vδ2 cells (%) is shown. (*N* = 4. Bars represent means ± SD. For each analyte, repeated-measures ANOVA with Geisser–Greenhouse correction and Tukey’s multiple comparison test was performed with individual variances computed for each comparison *P < .05.) **B**. ZA and heat-killed BCG-expanded Vδ2+ cells were isolated using MACS negative isolation and cultured overnight with 5 × 10^4^ CTFR-labelled target cells at a 3:1 effector:target cell ratio. THP-1 target cells were cultured overnight either with or without 50 µM ZA or 1 × 10^5^ CFU BCG prior to initiation of co-culture. >5000 CTFR+ cells were acquired using a BD LSR Fortessa X20 flow cytometer. Target cells were gated based on CTFR positivity. The specific killing was calculated by subtracting the frequency of dead target cells (as determined by live/dead red positive staining) in target alone conditions from the frequency of dead cells in co-culture conditions. (*N* = 12. Bars represent means ± SD repeated measures ANOVA with Geisser–Greenhouse correction and Tukey’s multiple comparison *P < .05.)

Having observed high levels of CD57 expression on ZA-expanded Vδ2+ cells, we sought to determine whether these cells express other markers that may be associated with an exhausted or functionally deficient phenotype. Flow cytometry was used to determine the frequency of expanded Vδ2+ cells expressing various checkpoint molecules after HKBCG and ZA expansion. ZA-expansion caused increased expression of PD1, TIM3, TIGIT, and LAG3 in a dose-dependent manner in Vδ2+ cells (Fig. 6A). Interestingly, whilst HKBCG expansion was associated with high levels of TIM3 and LAG3 expression, it was not associated with increased expression of PD1 which remained at levels of expression comparable to Vδ2+ cells expanded by concentrations of ZA as low as 0.5 µM. The functional implications of the dose-dependent increases in checkpoint molecule expression caused by ZA were investigated using a cytotoxicity assay. THP-1 cells were co-cultured with Vδ2+ cells expanded using different concentrations of ZA or HKBCG. Concordant with checkpoint molecule expression data, Vδ2+ cells expanded using higher concentrations of ZA were poorer at killing THP-1 cells compared to HKBCG expanded Vδ2+ cells and Vδ2+ cells expanded using low ZA concentrations (Fig. 6B). This provides further evidence that ZA-expanded Vδ2+ cells may be associated with a highly differentiated or exhausted phenotype.

### Intradermal BCG vaccination of rhesus macaques causes increases in circulating Vδ2^+^ cell frequency

**Figure 7:**
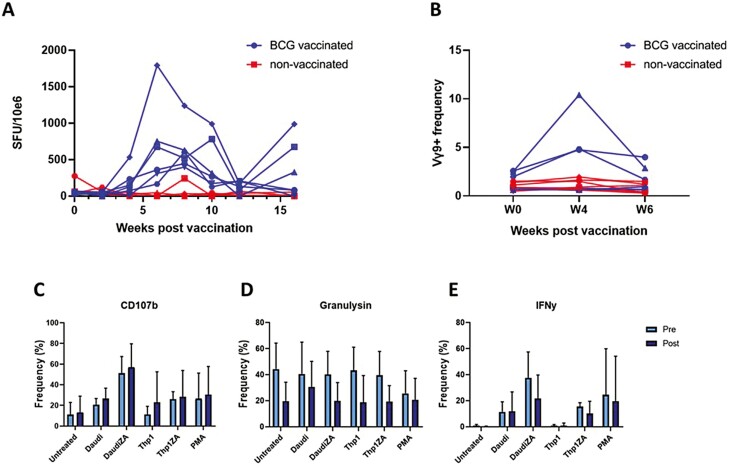
Phenotypic and functional characteristics of PBMCs obtained from repeated blood sampling of 6 BCG-vaccinated rhesus macaques (blue) and 6 non-vaccinated macaques (red). **A**. The IFN-γ response to BCG vaccination was measured using ELIspot. PBMCs were stimulated with 10 μg/mL purified protein derivative (PPD) (SSI, Copenhagen). Shown are numbers of IFN-γ spot forming units per 106 PBMCs. **B**. The frequency of Vγ9+ cells as a percentage of all CD3+ cells from 6 vaccinated individuals (blue) and 6 non-vaccinated individuals (red) was determined using flow cytometry. **C—E**. PBMCs from 6 BCG-vaccinated macaques collected pre- and 4 weeks post-BCG vaccination were co-cultured with the indicated target cells at an effector:target ratio of 10:1 for 4.5 h in the presence of anti-CD107b antibody, monensin, and brefeldin. **A**. Plots show frequencies of Vδ2+ cells expressing CD107b, granulysin, and IFN-γ. (*N* = 6. Bars represent means ± SD. Multiple paired *T*-tests with Sidak–Bonferroni correction for multiple comparisons.)

To determine the physiological relevance of the finding that BCG is capable of causing expansion of Vδ2^+^ cells with enhanced anti-tumour activity, 6 rhesus macaques were vaccinated intradermally with BCG and the frequency of Vδ2^+^ cells in their peripheral blood was measured 4- and 6-weeks post-vaccination. Due to well-documented cross-reactivity with non-human primate species, the 7A5 Vγ9 clone was selected for these assays over anti-Vδ2^+^ antibodies using the assumption that almost all circulating Vγ9^+^ cells co-express the Vδ2^+^ delta chain. Intradermal vaccination successfully induced BCG-specific responses in all vaccinated animals as determined using IFN-γ ELISpot which showed increased responsiveness to subsequent PPD stimulation in PBMCs of all vaccinated animals (Fig. 7A). The frequency of circulating Vγ9^+^ cells was elevated in a subset of vaccinated animals 4 weeks post-BCG vaccination before returning to baseline levels at 6 weeks (Fig. 7B). This suggests that delivery of BCG via the same route used as standard in human vaccination can cause an increase in Vδ2^+^ cell frequency, reminiscent of the results obtained from *in vitro* BCG treatment of human PBMCs (Fig. 1D).

PBMCs isolated from blood collected from macaques pre-vaccination and 4 weeks post-vaccination were co-cultured with ZA-pre-treated or untreated tumour cell lines for 4 h in the presence of Brefeldin A, monensin, and anti-CD107b as previously described for *in vitro*-expanded human Vδ2^+^ cells. Xenogeneic recognition of human tumour cell lines by macaques Vδ2^+^ cells occurred at low levels as expected based on the non-MHC-dependent mechanism of Vγ9Vδ2 TCR recognition. As in the results described with human Vδ2^+^ cells, macaque Vδ2^+^ cells degranulated and produced IFN-γ in response to human tumour cells lines, and the magnitude of these responses was elevated by pre-treatment of tumour cell lines with ZA (Fig. 7C–E). No significant differences were observed between pre-vaccination and post-vaccination Vδ2^+^ cells in terms of effector responses although a consistent but non-significantly lower level of granulysin expression was observed in Vδ2^+^ cells from post-vaccination samples. These results provide proof of the concept that these xenogeneic assays can be used to assess the effect of *in vivo* BCG vaccination on Vδ2^+^ cell anti-tumour responses in macaques through higher numbers of replicates are required to make conclusive statements about whether BCG vaccination can affect the anti-tumour potential of circulating Vδ2^+^ cells.

## Discussion

Meta-analysis of gene expression data from ~9000 tumours has shown that of all leukocyte subsets investigated, high γδ T-cell frequency correlated most closely with favourable disease outcomes [36]. This finding strongly suggests that γδ T cells are associated with anti-tumour immunity. Evidence in both this report and others shows that the circulating Vδ2^+^ subset of γδ T cells exhibits strong anti-tumour responses encompassing direct cytotoxic function and cytokine production [37–42]. Despite this data, clinical trials in which Vδ2^+^ cell-based interventions have been tested in various cancer types have yielded diverse and often disappointing results. The majority of trials aiming to harness the aforementioned anti-tumour potential of Vδ2^+^ cells *in vivo* have used either phosphoantigens or ZA to do so. Whilst proliferation and differentiation of Vδ2^+^ cells have been reported in some patients treated with ZA [18, 20], not all individuals receiving such treatments undergo measurable changes in Vδ2^+^ cell frequency or other characteristics [19] and few benefit from treatment as determined by disease progression. Clearly, novel methods of manipulating Vδ2^+^ cell anti-tumour functions are necessary to realize their potential in cancer therapy.

BCG was investigated in this study as a potential inducer of Vδ2^+^ cell anti-tumour function based on evidence that it can cause Vδ2^+^ cell expansions *in vivo* and induces tumouricidal effects in malignancies such as melanoma [29, 43, 44]. Results presented in this study show that *in vitro* BCG treatment of PBMCs leads to the proliferation of Vδ2^+^ cells which exhibit cytotoxic effects toward tumour cell lines. This may be associated with the well-documented ability of Vδ2^+^ cells to recognize both bacteria-derived phosphoantigens such as HMBPP (and other antigens such as mGLP) and endogenous pAgs like IPP which are overexpressed in some tumour cell lines. Previous work from our group has also shown that BCG treatment of PBMCs results in a coordinated response involving CD4^+^ T cells and myeloid dendritic cells (mDCs), which causes Vδ2^+^ cell activation and cytokine production [45]. Therefore, multiple mechanisms likely contribute to the activation of tumour-responsive Vδ2^+^ cells by BCG.

In the work presented in this report, we build on these observations, showing that BCG is capable not only of activation of Vδ2^+^ cells but also of promoting their expansion and inducing Vδ2^+^ cells with a sustained and enhanced ability to elicit both cytotoxic and cytokine responses to tumour cells. Moreover, we show that zoledronic acid expansion of Vδ2^+^ cells generated a population of cells with relatively less robust anti-tumour effector responses in terms of cytokine and cytolytic mediator production compared to BCG expansion.

The mechanisms underlying the improved anti-tumour priming of Vδ2^+^ cells after BCG expansion compared to ZA expansion remain obscure, but could depend on a number of factors including the effect of signalling via TLRs or other Vδ2^+^ PRRs induced by BCG concomitantly with HMBPP-dependent Vγ9Vδ2 TCR signalling; the effect of cytokines generated by PBMCs in response to BCG stimulation priming differential γδ T-cell responses; and the expansion of Vδ2^+^ cell populations with differing TCR repertoires caused by ZA and BCG expansion resulting in different responses to target cells. Additionally, it could be the case that ZA causes detrimental effects on Vδ2^+^ cell function for example by inhibition of mevalonate metabolism impeding anti-tumour responses of ZA-expanded Vδ2^+^ cells.

The suggestion that cytokine production by PBMCs after BCG stimulation could affect subsequent functional characteristics of Vδ2^+^ cells is based on both observations from our own laboratory that CD4+ T-cell and mDC-mediated IL-12p70 production supports Vδ2^+^ cell activation and recent evidence from García-Cuesta et al. supporting the idea that BCG-induced cytokine responses by PBMCs lead to enhanced cytotoxic effects in lymphocyte subsets [45, 46]. The authors demonstrated that BCG treatment of PBMCs caused the proliferation of CD56^bright^ NK cells which were primed to elicit more potent degranulation responses toward tumour target cells. It was suggested that this priming effect was mediated by soluble factors produced by PBMCs in response to BCG. Among others, the authors showed that IL-12p70 IL-1β and IL-23 were produced by BCG stimulated PBMCs, all of which have been shown to modify the cytokine production profile of TCR stimulated Vδ2^+^ cells [47, 48].

Another potential explanation for the observed differences in effector responses between ZA- and BCG-expanded Vδ2^+^ cell populations could be derived from differential skewing of their clonal diversity. Hoft *et al*. have shown the differential distribution of Vδ2^+^ cell clonotypes in pAg-expanded, compared to mycobacteria-expanded, Vδ2^+^ cells [49]. A narrower range of clonotypes arose after expansion with mycobacteria, which was associated with an enhanced ability to limit mycobacterial growth upon subsequent co-culture with M.tb-infected cells. In subsequent publications, the group used nuclear magnetic resonance, and mass spectrometry to identify 6-*O*-methylglucose-containing lipopolysaccharides (mGLP) as the major component of mycobacteria necessary to induce expansion of a population of Vδ2^+^ cells with the ability to inhibit mycobacterial growth [50]. Vδ2^+^ cells expanded using enriched mGLP elicited more potent cytokine responses and produced higher levels of granzyme A in response to BCG-infected DCs compared to HMBPP-expanded or M.tb whole lysate-expanded Vδ2^+^ cells. This is particularly interesting in the context of data presented in this study which shows that BCG-expanded Vδ2^+^ cells produced higher concentrations of granzyme A in response to THP-1 and THP-1(ZA) target cells compared to ZA-expanded.

In this study, we document the decreased levels of degranulation of ZA expanded Vδ2^+^ cells as well as their altered cytokine production and increased proliferation. One potential explanation for the detrimental effects of ZA on Vδ2^+^ cells is the potential of ZA to drive Vδ2^+^ cells toward an exhausted or senescent phenotype. This is supported by the unique expression of CD57 on ZA-expanded cells and the increased expression of immune checkpoint molecules widely regarded as markers of T-cell exhaustion. NBPs have been documented to decrease circulating γδ T cells with repetitive stimulation resulting in progressive exhaustion [51]. On the other hand, it has been shown that bacterial infection with *Salmonella enterica* generates Vδ2^+^ immunity with a high level of prolonged expansion and lack of anergy due to overproduction of HMBPP which is more reminiscent of the response to bacterial infection [11]. The finding of a cluster of CD57 expressing Vδ2^+^ cells following expansion with ZA suggests a potential for these cells to be senescent. CD57 has been documented to be expressed on T cells and NK cells in the final stages of maturation with reduced ability to proliferate but retention of cytotoxic function [52, 53]. In Vδ2^+^ cells, the marker was associated with shorter telomere length suggesting a population that had undergone higher levels of proliferation [35]. The observation of a CD57 expressing population unique to ZA-expanded Vδ2^+^ cells could define a subset of senescent cells which could underlie the differences observed between BCG and ZA- expanded Vδ2^+^ cells in terms of their cytokine production.

Recent data from Ryan *et al*., show that Vδ2^+^ cells with different dominant phenotypic profiles preferentially produce different cytolytic mediators in response to tumour cells [34]. Specifically, different Vδ2^+^ cell phenotypes post-expansion preferentially produced either granzyme B or granzyme K in responses to tumour cell lines, differentially affecting their ability to lyse a range of tumour cell lines. This raises the possibility that phenotypic skewing of BCG-expanded Vδ2^+^ cells favoured cytotoxic responses toward cell lines used in the research presented in this study. Another recently appreciated implication of enhanced granulysin production by BCG-expanded Vδ2^+^ cells is that granulysin could have a role in the activation and recruitment of the adaptive immune system [54]. More specifically, we have recently published data showing that granulysin, released by Vδ2+ cells, can induce migration and maturation of immature DCs, suggesting the potential for BCG-expanded Vδ2^+^ cells to more effectively contribute to enhancing tumour antigen presentation by DCs [33]. High concentrations of granulysin chemoattracts immature DCs, which can then be matured, in part by granulysin, and mature DCs migrate to lower concentrations. Moreover, the *in vivo* studies presented here show a reduced granulysin content of activated Vδ2^+^ cells 4 weeks post-BCG vaccination, possibly suggesting the release of this molecule over the 4 weeks between vaccination and testing. In our previous paper, we show that granulysin secretion increases from expanded Vδ2^+^ cells for over 9 days suggesting long-term release of this molecule occurs [33].

Observations that intradermal BCG vaccination of rhesus macaques also induced Vδ2^+^ cell proliferation suggest that this method of BCG delivery could be sufficient for the generation of the cytotoxic BCG-responsive Vδ2^+^ cell populations observed in the *in vitro* experiments performed. Supporting this assertion, Yang et al. have shown that intradermal vaccination with BCG followed by subsequent intralesional administration of BCG in melanoma is associated with regression of lesions and concurrent increases in Vδ2^+^ cell presence in lesions that visibly regressed [29]. Interestingly, the authors showed that non-BCG injected lesions proximal to the injected lesions also regressed and that this regression was associated with the presence of γδ T cells. Future work will aim to address this question by further characterizing the responses of *in vivo* BCG-expanded Vδ2^+^ cells towards tumour cells.

Overall, the results presented in this report represent novel evidence supporting the principle that BCG could be used therapeutically to prime anti-tumour Vδ2^+^ cell responses. Specifically, results support the assertion that BCG could promote the expansion of Vδ2^+^ cells with superior cytolytic and cytokine-producing potential compared to ZA-mediated expansion which potentially results in cells with a more senescent or exhausted phenotype. Moreover, the results presented show that ZA-treatment of tumour cell targets increases their susceptibility to BCG-expanded Vδ2^+^ cell lysis. Together these findings argue for possible treatment modalities in which priming doses of BCG are used to promote Vδ2^+^ cell activation and expansion followed by ZA treatment, ideally at tumour site to enhance the targeting of Vδ2^+^ anti-tumour responses.

## Supplementary data

Supplementary data is available at *Clinical and Experimental Immunology* online. 

**Supplemental figure 1:** 5 × 10^5^ THP-1 cells were infected for 24 h with GFP+BCG. **A**. THP-1 cells were infected with 5 × 10^5^ CFU GFP + BCG and stained using DAPI. Imaging was conducted using a Nikon A1R with 40× CFI S Plan Fluor ELWD objective; 405 nm laser detected DAPI fluorescence is displayed in blue and 488 nm laser detected GFP fluorescence is displayed in green. A Z-stacked series of images running from top left to bottom right is displayed, with 0.5µm intervals between images. **B**. GFP transfected BCG was cultured overnight with 1 × 10^5^ THP-1 cells at a range of CFU values. The gating strategy displayed in Fig. 3A was applied to determine the percentage of dead THP-1 cells after culture based on the frequency of Zombie NIR positive cells. The plot is representative of two experimental repeats.

uxac032_suppl_Supplementary_Figure_S1

## Data Availability

The data underlying this article will be shared on reasonable request to the corresponding author.
