# Supplementary figures and images for: Bacillus Calmette-Guerin (BCG) induces superior anti-tumour responses by Vδ2+ T cells compared with the aminobisphosphonate drug zoledronic acid
Source: Clin Exp Immunol. 2022 Apr 11;208(3):301–15. doi: 10.1093/cei/uxac032 (PMC9226146; doi:10.1093/cei/uxac032)

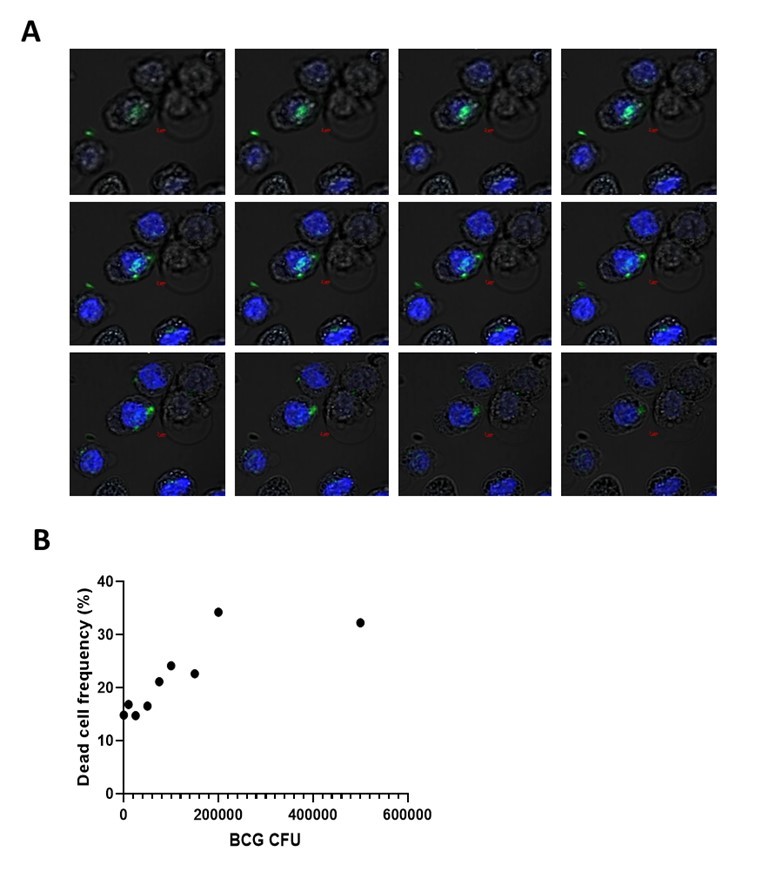

Supplement: uxac032_suppl_Supplementary_Figure_S1 [file uxac032_suppl_supplementary_figure_s1.jpeg]
